# 

**Published:** 1876-04

**Authors:** 


### ANNOUNCEMENTS FOR THE MONTH.

**MONDAYS.** SOCIETIES.

*Mondays, Apr.* 3 and 17—Chicago Med. Society, regular meetings at the Washingtonian Home, 8 P. M.

*Mondays, Apr.* 10 and 24 — Chicago Society of Physicians and Surgeons, regular meetings at Grand Pacific, 8 P. M.

CLINICS. *Every Monday.*

At Eye and Ear Infirmary, (Peoria and Adams Sts.) 2 P. M.—Prof. Holmes.
At Chicago College, 2 P. M. *Gynecological*—Prof. Merriman.
At Central Dispensary (239 W. Van Buren St.), 2 P.M., *Gynecological*—Dr. Adolphus; 3 P.M., *Medical*—Dr. Bridge.
At Mercy Hospital, 2 P. M., *Medical*—Prof. Johnson.

**TUESDAYS.** SOCIETIES.

*Tuesday, Apr.* 11—Academy of Sciences, regular meeting, 8 P. M. (263 Wabash Ave).
*Tuesday, Apr.* 25—Medico-Historical Society, annual meeting at the Tremont House.

CLINICS. *Every Tuesday.*

At County Hospital, 2 P. M., *Medical*—Prof. Bevan; 3 P. M., *Surgical*—Prof. Bogue.
At Chicago College, 2 P. M., *Gynecological*—Prof. Roler.
At Mercy Hospital, 2 P. M., *Medical*—Prof. Hollister.

**WEDNESDAYS.** CLINICS. *Every Wednesday.*

At County Hospital, 2 P. M., *Ophthalmological*—Dr. Montgomery; 3 P. M., *Gynecological*—Prof. Quine.
At Chicago College, 2 P. M., *Gynecological*—Prof. Nelson.
At Mercy Hospital, 2 P. M., *Surgical*—Prof. Andrews.
At Central Dispensary, 2 P. M., *Medical*—Dr. Bridge.
At St. Luke's Hospital, commencing at 1.30 P. M., *Surgical*—Dr. Owens.

**THURSDAYS.** CLINICS. *Every Thursday.*

At Chicago College, 2 P. M., *Gynecological*—Prof. Merriman.
At Central Dispensary, 2 P. M., *Diseases of Chest*—Dr. Ingals.
At Mercy Hospital, 2 P. M., *Medical*—Prof. Johnson.

**FRIDAYS.** SOCIETIES.

*Friday, Apr.* 14—State Microscopical Society of Illinois, regular meeting at the Academy of Sciences, 8 P. M.

CLINICS. *Every Friday.*

At County Hospital, 2 P. M., *Medical*—Prof. Bevan; 3 P. M., *Surgical*—Prof. Bogue.
At Chicago College, 2 P. M., *Gynecological*—Prof. Roler.
At Mercy Hospital, 2 P. M., *On Diseases of Eye and Ear*—Prof. Jones.
At Central Dispensary, 2 P. M., *Gynecological*—Dr. Adolphus.

**SATURDAYS.** CLINICS. *Every Saturday.*

At Rush College, 2 P. M., *Surgical*—Prof. Gunn; 3 P. M., *Diseases of the Brain and Nervous System*—Dr. Hay.
At Chicago College, 2 P. M., *Gynecological*—Prof. Nelson; *Surgical*—Prof. Andrews; 3 P. M., *Medical*—Prof. Davis.

☞ At all the above Clinics visiting regular practitioners are, we believe, admitted.

